# Fusidic acid resistance among clinical isolates of methicillin-resistant *Staphylococcus aureus *in a Taiwanese hospital

**DOI:** 10.1186/1471-2180-11-98

**Published:** 2011-05-12

**Authors:** Chih-Ming Chen, Mei Huang, Huei-Fen Chen, Se-Chin Ke, Chia-Ru Li, Jen-Hsien Wang, Lii-Tzu Wu

**Affiliations:** 1 Division of Infectious Disease, Department of Internal Medicine, Tungs' Taichung MetroHarbor Hospital, Taiwan; 2 Division of Infectious Disease, Show Chwan Memorial Hospital, Taiwan; 3 The Institute of Medical Science and Department of Microbiology, China Medical University and Hospitals, Taichung, Taiwan; 4 Infection Control Office, Tungs' Taichung MetroHarbor Hospital, Taiwan; 5 Department of Medical Research, Tungs' Taichung MetroHarbor Hospital, Taiwan; 6 Department of Internal Medicine and Section of Clinical Microbiology, China Medical College-Hospital, Taichung, Taiwan; 7 Department of Laboratory Medicine, China Medical College-Hospital, Taichung, Taiwan

**Keywords:** fusidic acid, MRSA, *fusA*, *fusB*, *fusC*

## Abstract

**Background:**

The prevalence of resistance to fusidic acid of methicillin-resistant *Staphylococcus aureus *(MRSA) was increased each year in a Taiwan hospital. Thirty-four MRSA clinical isolates collected in 2007 and 2008 with reduced susceptibility to FA were selected for further evaluation the presence of resistance determinants.

**Results:**

The most common resistance determinant was *fusC*, found in 25 of the 34 MRSA isolates. One of the 25 fusidic acid-resistant MRSA harboured both *fusB *and *fusC*, which is the first time this has been identified. Mutations in *fusA *were found in 10 strains, a total of 3 amino-acid substitutions in EF-G (*fusA *gene) were detected. Two substitutions with G_556_S and R_659_L were identified for the first time. Low-level resistance to fusidic acid (MICs, ≤ 32 μg/ml) was found in most our collection. All collected isolates carried type III SCC*mec *elements. MLST showed the isolates were MRSA ST239. PFGE revealed nine different pulsotypes in one cluster.

**Conclusions:**

Our results indicate that the increase in the number of fusidic acid resistant among the MRSA isolates in this hospital is due mainly to the distribution of *fusC *determinants. Moreover, more than one fusidic acid-resistance mechanism was first detected in a same stain in our collection.

## Background

The frequently-encountered multi-antibiotic resistance of MRSA has become a major health problem [1,2]. The prevalence of MRSA isolates, most of which are health care associated, has slowly increased since 1982, and the appearance and increasing incidence of community-associated MRSA infections has been documented. Globally, methicillin resistance among nosocomial *S. aureus *isolates is common [3,4].

Fusidic acid has been used to treat infections with *S. aureus *for over 35 years. It is usually used in combination with agents such as vancomycin or rifampin in the treatment of systemic infections caused by MRSA [5]. Fusidic acid inhibits protein synthesis by blocking the elongation of the nascent polypeptide chain through binding to EF-G on the ribosome and preventing the dissociation of EF-G⋅GDP from the ribosome [6,7]. The frequency of fusidic acid resistance is not very high; however, the emergence of clinical staphylococcal species that are resistant to fusidic acid has been reported [8-11].

The primary mechanism of fusidic acid resistance in *S. aureus *relates to mutations in *fusA*, the gene that encodes the ribosomal translocase and translation elongation factor EF-G [12,13]. More than 30 different amino acid substitution mutations in *fusA *have been identified [12,14,15]. Subsequently, resistance in natural isolates may also result from the horizontal acquisition of *fusB*, a poorly characterized plasmid-mediated resistance mechanism [13]. The gene *fusB *is usually carried by a 21-kb plasmid, pUB101 [16], however, it can also be chromosomal [17]. The *fusB *gene encodes an inducible protein that protects an in vitro translation system against the inhibitory action of fusidic acid [8]. Recently, two *fusB *homologues, designated *fusC *and *fusD*, have been identified in the chromosome of clinical isolates of *S. aureus *and *S. saprophyticus*, respectively [18]. In addition, fusidic acid-resistant small-colony variants (SCVs) of *S. aureus *with mutations in *rplF *have been designated as FusE mutants [14]. Although frequencies of resistance to fusidic acid have remained generally low, each of these mechanisms has multiple genetic causes, and emerging resistance is a problem that could limit the therapeutic options available for treatment of staphylococcal infections [19].

In this study, a series of MRSA clinical isolates recovered at a regional teaching hospital in middle Taiwan showing fusidic acid MIC ≥ 2 μg/ml. The high distribution of fusidic acid resistance determinants *fusC *was confirmed in MRSA. In addition, different fusidic acid resistance determinants-containing in one isolate was also demonstrated.

## Methods

### Bacterial isolates

From April 2007 to January 2008, 34 clinical isolates of MRSA with fusidic acid resistance were recovered from 34 different patients at Tungs' Taichung MetroHarbor Hospital (TTMHH), a 1405-bed regional teaching hospital in central Taiwan. *S. aureus *ATCC 29213 and NCTC 8325 have consistently been used as a quality control strain and Pulsed Field Gel Electrophoresis (PFGE) standard strain, respectively. Luria-Bertani (LB) agar and LB broth were used for bacterial growth at 37°C with aeration. Mueller-Hinton agar was used for all determinations of minimum inhibitory concentrations (MICs). All isolates were identified on the colony morphology, Gram's stain, a positive catalase reaction and/or results obtained with the phoenix system (BD Diagnostic Systems, Sparks, MD, USA) and frozen at -80°C until used.

### Antimicrobial susceptibility tests

MICs of different antimicrobial agents were determined using the Phoenix Automated Microbiology System (BD Diagnostic Systems, Sparks, MD) and interpreted according to the criteria provided by the Clinical and Laboratory Standards Institute (CLSI). Fusidic acid susceptibility was screened by the disk diffusion method with 10 μg fusidic acid containing disks. The interpretive criterion of susceptibility was an inhibition zone ≥ 22 mm in diameter. Fusidic acid MICs were further determined by an agar dilution method following the CLSI guidelines, and susceptibility was categorized using the European Committee for Antimicrobial Susceptibility Testing (EUCAST)/British Society of Antimicrobial Chemotherapy (BSAC) criteria (susceptible, MIC < 2 μg/ml; resistant, MIC ≥ 2 μg/ml). The testing MIC range of fusidic acid was 0.12-512 μg/ml.

### DNA manipulation and PCR

Total DNA from three to five isolated colonies was prepared using a Wizard genomic DNA preparation kit (Promega, Madison, WI) with 0.5 mg/ml of lysostaphin and 0.3 mg/ml of RNase for the lysis step. The multiplex PCR assay for *fusB *and *fusC *used oligonucleotide primers BF (5'-CTATAATGATATTAATGAGATTTTTGG), BR (5'-TTTTTACATATTGACCATCCGAATTGG), CF (5'-TTAAAGAAAAAGATATTGATATCTCGG), and CR (5'-TTTACAGAATCCTTTTACTTTATTTGG) to generate amplicons of 431 and 332 bp from the *fusB *and *fusC *genes, respectively. The cycling conditions consisted of an initial denaturation step (94°C for 3 min), followed by 25 cycles of 94°C (30 s), 57°C (30 s) and 72°C (45 s) [20]. For further identification of the *fus*B and *fus*C genes, primers FusB-R (5'-ACAGGATCCATTTTCACAAACATAGT) and FusB-F1(5'-AGGGATCCCATATTTAAAGCTATTG) were used to generate an amplicon comprising the 642 bp *fusB *with 122 bp of upstream DNA [8], and primers sas0043U (5'-GTAGGATCCATTGGGAATGATAAATAGTGA) and sas0043L (5'-TTTGGATCCATCGATTAAGAGTGAGGTACA) were used to generate a 2.5 kb amplicon with *fus*C [18]. The *fusA *gene was PCR-amplified using oligonucleotide primers rpsU and tufL and sequenced with these and three additional primers (AintS1, 5'-TAAGGGTCAGTCATAACTTT; AintS2, 5'-TTCAAAAACAAAGGTGTTCA; and AintS3, 5'-ATGTATTCACGAGGAAC) [20]. The PCR products were electrophoresed in 1.5% agarose gels and visualized under ultraviolet light. The PCR products were then purified with a commercial kit and both strands of the amplicons were sequenced on an ABI PRISM 370 automated sequencer (PE Applied Biosystems, Franklin Lakes, NJ). Sequence analyses were performed online at the National Center for Biotechnology Information website (http://www.ncbi.nlm.nih.gov).

### Southern blot hybridization

DNA samples were digested by *Eco*R1 and analyzed by electrophoresis at 30 V for 2 h in a 1% w/v agarose gel. The gel was denatured in a solution of 0.5 M NaOH and 1.5 M NaCl, neutralized in 0.5 M Tris-HCl (pH 7.5) and 1.5 M NaCl on Whatman filter paper (Maidstone, UK), and finally saturated with 10% w/v SDS (15 min for each step). DNA was transferred to a positively charged nylon membrane (Boehringer Mannheim, Mannheim, Germany) using an electrophoretic transfer cell (Bio-Rad Laboratories, Hercules, CA). A probe for *fusC *was prepared by randomly labelling the 2.5 kb PCR product of *fusC *with digoxigenin using a commercial kit (Roche Diagnostics, Mannheim, Germany) according to the manufacturer's instructions. The *fusC *gene for the hybridization probe was amplified using oligonucleotide primers fusCU 5'-GAGGAATATCATATGAATAAAATAGAAGTGTA and fusCL 5'-AGAGTGGATCCCAAAATATAACAACCCTGATC [18].

### SCCmec typing by PCR

The presence of *mecA *was determined using the primers MR1 5'-GTGGAATTGGCCAATACAGG and MR2 5'-TGAGTTCTGCAGTACCGGAT, which were used to PCR-amplify a 1,339 bp internal fragment of the gene [21]. PCR was carried out for 30 cycles of 1 min at 95°C, 1 min at 55°C, and 2 min at 72°C. Characterization of SCC*mec *elements was performed by multiple PCR as previously described [22].

### PFGE and multilocus sequence typing (MLST)

Genotyping of *S. aureus *strains was conducted by macrorestriction of bacterial DNA followed by PFGE separation of the resulting fragments. Whole chromosomal DNA of the clinical isolates embedded in agarose gel plugs (FMC Bioproducts, Philadelphia, PA) were treated with proteinase K and *Sma*I restriction endonuclease according to the manufacturer's recommendations (New England Biolabs, Ipswich, MA). PFGE and DNA fingerprints analysis were performed as described previously [23]. The isolates were also analyzed by MLST as described previously [24].

### Plasmid curing

The clinical isolate with pUB101-like plasmid was subjected to elevated temperature-mediated plasmid elimination by sequential passages in LB (approximately 100 cells into 100 ml) at 43°C with shaking for about 30 generations. Cured strains were diluted and plated on LA plates (LB plus 1% agar; Merck, Darmstadt, Germany) to obtain single colonies. Loss of cadmium resistance was screened by replica plating at 37°C [25]. Loss of the plasmid was confirmed by loss of unselected phenotypic traits (ampicillin resistance) and by PCR of cadXD [15].

### Ethics

This study was reviewed by the Institutional Review Board (IRB) of the TTMHH and it was decided not to constitute the research involving human subject. An exemption certificate was issued by the IRB to attest this fact.

## Results

### Isolates and susceptibility tests

The sources of the 34 fusidic acid-resistant MRSA isolates included sputum (n = 9), pus (n = 16), blood (n = 5), urine (n = 2), ascites (n = 1), and tip of a central venous catheter (n = 1) (Table 1). All 34 clinical isolates were analyzed in more detail with regard to their antibiotic resistance profiles, and they were all susceptible to vancomycin, teicoplanin, quinupristin-dalfopristin, linezolid, and nitrofurantoin. The MICs for fusidic acid (2-64 μg/ml) were low to moderate level resistance phenotype. All isolates were uniformly resistant to penicillin, ampicillin, oxacillin, clindamycin, erythromycin, ciprofloxacin and gentamicin. The susceptible rates and MIC ranges of other antibiotics were as follows: rifampin 91%; chloramphenicol 88%; moxifloxacin 6%; levofloxacin 3%; tetracycline 3%; and trimethoprim-sulfamethoxazole 3%. The study results revealed that fusidic acid-resistant *S. aureus *was resistant to nearly all tested antibiotics except for vancomycin, teicoplanin, linezolid, nitrofurantoin, quinupristin-dalfopristin, chloramphenicol, and rifampin.

**Table 1:** Characteristics and mechanisms of the 34 fusidic acid-resistant MRSA clinical isolates

Isolate	Specimen	FA MIC (μg/ml)	VAN MIC (μg/ml)	LZD MIC (μg/ml)	OXA MIC (μg/ml)	RIF MIC (μg/ml)	*fusC*	*Polymorphism(s)* *in *EF-G	PFGE patterns
1	Pus	8	1	1	>2	< = 0.5	-	H_457_Y	A1
2	Pus	32	1	2	>2	< = 0.5	+	-	A1
3	Pus	8	1	1	>2	< = 0.5	+	-	A1
4	Sputum	16	1	2	>2	< = 0.5	+	H_457_Y	A1
5	Sputum	32	2	2	>2	>2	+	-	A1
6	Pus	16	1	1	>2	>2	+	-	A2
7	Pus	8	1	1	>2	< = 0.5	+	-	A3
8	Sputum	16	1	1	>2	< = 0.5	+	-	A3
9	Pus	16	1	1	>2	< = 0.5	-	G_556_S	A3
10	Sputum	16	1	1	>2	< = 0.5	-	H_457_Y, G_556_S	A3
11	Ascites	8	1	1	>2	< = 0.5	-	H_457_Y	A3
12	Pus	64	2	2	>2	< = 0.5	+	-	A3
13	Sputum	64	2	2	>2	< = 0.5	-	H_457_Y	A3
14	Pus	16	1	1	>2	< = 0.5	+	-	A3
15	Blood	4	1	1	>2	< = 0.5	+	-	A3
16	Pus	8	1	1	>2	< = 0.5	+	-	A3
17	Blood	8	1	1	>2	< = 0.5	+	-	A3
18	Blood	16	1	1	>2	< = 0.5	+	-	A3
19	Blood	16	1	1	>2	< = 0.5	+	-	A3
20	Pus	2	2	1	>2	< = 0.5	+	-	A3
21	Urine	2	2	2	>2	< = 0.5	-	H457Y, G556S	A3
22	Sputum	2	2	2	>2	< = 0.5	+	-	A3
23	Pus	16	2	1	>2	>2	-	H457Y	A4
24	Pus	2	1	1	>2	< = 0.5	+	-	A5
25	Urine	16	1	1	>2	< = 0.5	+	-	A6
26	CVP tip	8	1	2	>2	< = 0.5	+	-	A6
27	Pus	2	2	2	>2	< = 0.5	+	-	A6
28	Sputum	16	1	2	>2	< = 0.5	+	-	A7
29^a^	Pus	8	1	2	>2	< = 0.5	+	-	A8
30	Sputum	16	1	2	>2	< = 0.5	+	-	A9
31	Pus	16	1	2	>2	< = 0.5	-	H457Y, R659L	A9
32	Sputum	8	1	2	>2	< = 0.5	+	-	A9
33	Blood	16	1	1	>2	< = 0.5	-	G556S	A9
34	Pus	2	2	2	>2	< = 0.5	+	-	A9

FA, fusidic acid; VAN, vancomycin; LZD, linezolid; OXA, oxacillin; RIF, rifampin

^a ^nonsense mutation in *fusC *(S175 was encoded by TAA rather than TCA)

### Genetic basis of resistance to fusidic acid: fusB and fusC

The genetic basis for resistance to fusidic acid in the isolates was determined by a multiplex PCR assay capable of detecting both the 431 bp *fus*B and 332 bp *fus*C genes [20]. Twenty-five of the 34 isolates (73.5%) were found to harbour the gene encoding *fus*C and one (isolate 32) among the 25 isolates also harboured the gene encoding *fus*B. Furthermore, using plasmid DNA of isolate 32 as a template, PCR with FusB-specific primers FusB-R1 and FusB-F1 and subsequent sequence analysis of the 764 bp PCR product confirmed the 100% identity of the *fus*B gene from plasmid pUB101. A curing study revealed that both the *cad*XD and *fus*B genes were plasmid encoded, and that *fus*C remained in the plasmid cured isolate 32. The MIC of fusidic acid for isolate 32 was 8 μg/ml after curing of the plasmid.

**Figure 1:**
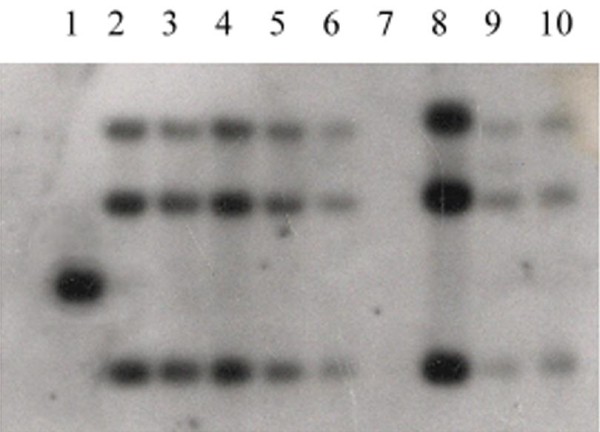
**Southern hybridization of *fusC***. Detection of *fusC *by Southern hybridization in eight representatives of clinical fusidic acid-resistant *S. aureus *isolates that did not harbour *fusB *or resistance polymorphisms in *fusA*. Lane 1: 2.5-kb PCR *fus*C fragment from strain 2 as the positive control. Lanes 2-6 and 8-10: strains 3, 6, 15, 18, 24, 28, 29 and 34, respectively. Lane 7: strain 23 without the *fus*C gene. All total DNA was *Eco*RI-digested.

The full-length *fus*C gene was identified by PCR and sequenced in isolates 4, 24, 29, 30, and 32. The alignment of the amino acid sequences deduced from these isolates 4, 24, 30, and 32 *fus*C DNA sequences revealed 100% identity with FusC protein of *S. aureus *MSSA476 [18]. However, *fusC *from isolate 29 carried a nonsense mutation (S175 was encoded by TAA rather than TCA) that produced a change from fusidic acid resistance (MIC = 8 μg/ml) to fusidic acid susceptibility (MIC < 0.125 μg/ml) following two non-selective subcultures. The other isolates were screened for the presence of the *fus*C gene by Southern hybridization, and all tested isolates were positive for *fus*C (Figure 1).

### Detection of fusA gene mutations

PCR amplification and complete sequencing were performed to detect *fusA *gene mutations in the 34 isolates (Table 1). Five isolates possessed a mutation in H_457_Y, two isolates (isolates 9 and 33) exhibited a G_556_S mutation, and two isolates (isolates 10 and 21) harboured mutations in H_457_Y and G_556_S. In addition, isolate 31 possessed a mutation in H_457_Y and R_659_L. Single amino acid substitutions were found in seven isolates, and two amino acid substitutions were found in the other three. This is the first time that two different amino acid substitutions, G_556_S and R_659_L, have been reported in *fus*A gene mutations. Furthermore, one isolate (isolate 4) was encoded with *fusC *and *fus*A gene mutation. In this study, the most common amino acid substitution H_457_Y did not result in a high level of fusidic acid resistance (MIC ≥ 128 μg/ml).

### Molecular epidemiological analysis

**Figure 2:**
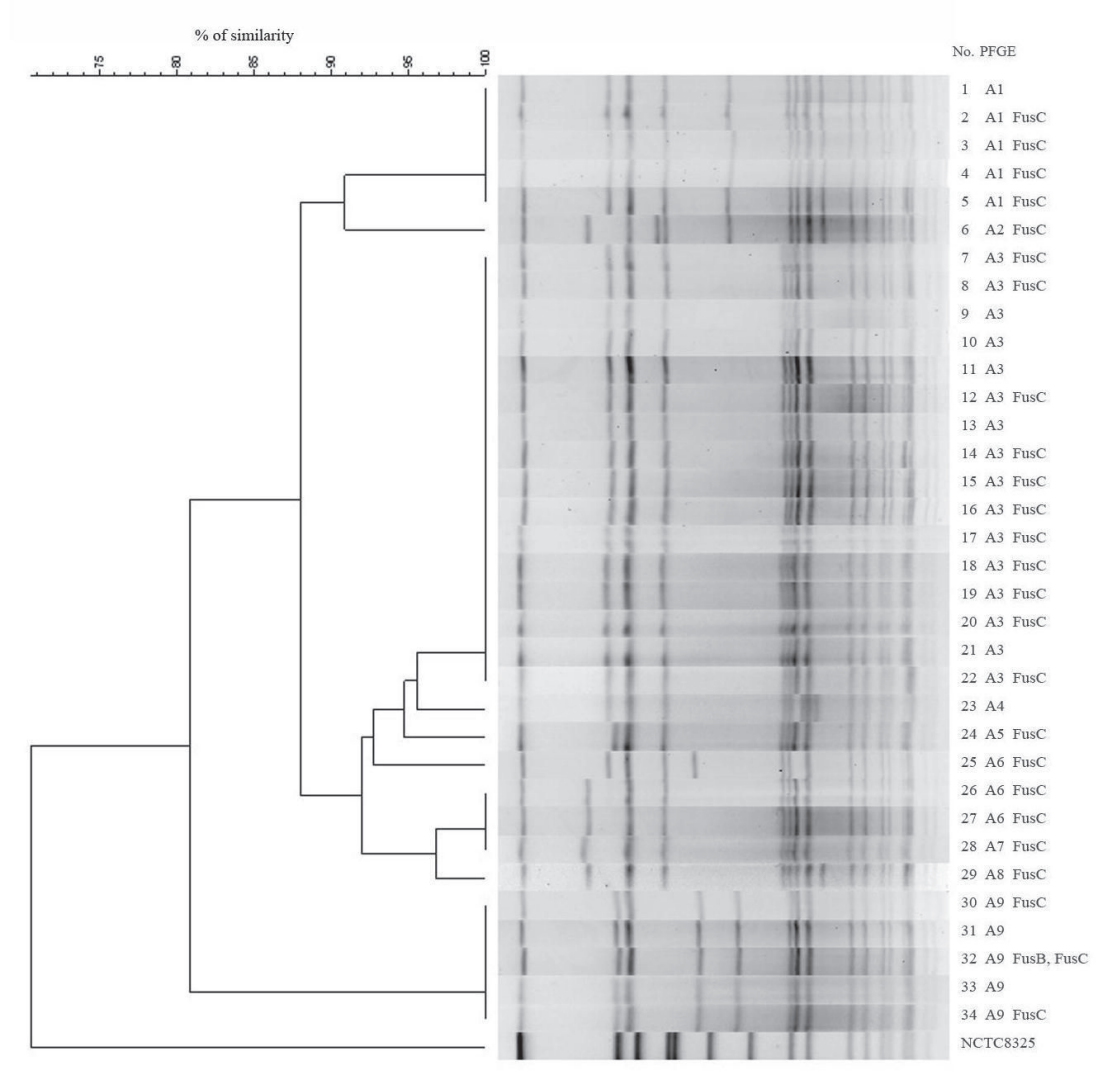
***Sma*I PFGE patterns of the 34 clinical fusidic acid-resistant *Staphylococcus aureus *isolates**. PFGE patterns analysis of these 34 isolates revealed nine distinct pulsotypes (A1-A9) that were classified into one cluster.

All 34 isolates included in this study met the criteria of being health care associated. The genotype analyses and their frequencies are shown in Table 1. Only one defined MLST type (ST239) was evident. All 34 isolates carried SCC*mec *type III elements. PFGE patterns of *Sma*I macrorestriction fragment analysis of these 34 isolates revealed nine distinct pulsotypes (A1-A9) that were classified into one cluster (> 80% similarity) (Figure 2). The results of PFGE patterns are summarized in Table 1.

## Discussion

Previous studies of fusidic acid-resistance in clinical isolates have mostly focused on methicillin-susceptible *S. aureus *(MSSA) and other staphylococci [17,20,26]. Chen et al. recently reported that the prevalence of fusidic acid-resistance determinants was quite different between MRSA and MSSA groups [27]. In northern Taiwan collections, the *fusA *mutations were the major determinant (84%) followed by *fus*C with 16% fusidic acid-resistance in MRSA isolates [27]. In the present study based in central Taiwan, we found that the fusidic acid-resistant predominant determinant in MRSA was a high prevalence of *fus*C with 74% in clinical isolates. Furthermore, one isolate carried the *fus*B determinant on the plasmid and *fus*C determinant on the chromosome in a clinical fusidic acid-resistant *S. aureus *isolate. The FusC protein has a 45% amino acid similarity to FusB. The *fusC *gene was originally identified in the genome sequence of *S. aureus *MSSA476, and has been reported in fusidic acid-resistant *S. intermedius *and *S. epidermidis *[18,20]. In most European collections, *fusC *has been shown to be responsible for resistance to fusidic acid in all *S. aureus *strains examined that do not carry *fusB *or resistance mutations in *fusA *[17,18]. Moreover, the *fusB *gene has only been detected in MSSA, not in MRSA in most clinical collections in Taiwan [27]. Therefore, the present study shows the spread of *fusC *in Taiwan and for the first time demonstrates the presence of both *fusB *and *fusC *in a MRSA clinical isolate.

The most common mutation in *fusA *that conferred resistance to fusidic acid was the substitution H_457_Y in our study (Table 1). We reviewed the English literature and did not find any reports of two amino acid substitutions in EF-G of G_556_S and R_659_L relative to the resistance of fusidic acid. Mutations in EF-G are associated with fitness cost in the fusidic acid-resistance of *S. aureus *in vitro and in vivo [12,14]. The resistance mutations with amino acid substitutions occur mostly in structural domain III of EF-G, but some occur in domains I and V [28,29]. We identified a novel substitution present in fusidic acid-resistant *S. aureus *(isolates 9 and 33), which conferred an identical resistance mutation in *fusA *(G_556_S). The two isolates exhibited resistance to fusidic acid with MIC = 16 μg/ml and carried neither *fusB *nor *fusC*. In addition, substitution G_556_S was found in isolates 10 and 21 and was accompanied by mutations in *fusA *(H_457_Y). Another novel substitution amino acid substitution R_659_L located in domain V of EF-G was found to be accompanied with *fusC *mutations in our study. The role of this newly found amino acid substitution in *fusA *on the level of resistance is unknown and needs further investigation. Of the 34 isolates that were studied completely, isolate 4 harboured *fusC *and a resistance mutation in *fusA *(H_457_Y). This indicates that the fusidic acid-resistance in these MRSA clinical isolates had multiple genetic lineages.

The isolates with *fusB *and *fusC *determinants usually displayed higher level resistance to fusidic acid (> 16 μg/ml) [8,17]. The MICs of fusidic acid in our collections carrying *fusC *ranged from 2-64 μg/ml. It is not clear the reason why in non-selective subcultures, isolate 29 with one mutation site of the *fusC *gene lost the resistance to fusidic acid. We hypothesized that the mutation may result in FusC truncated after amino acid 174, and thus isolate 29 became susceptible. In this study, the single-amino-acid substitutions in EF-G substitution did not result in a high level fusidic acid resistance which is similar to previous report in MRSA strains belonging to CC8, H_457_Y mutation was associated with MIC of 64 μg/L and H_457_Q was associated with MIC of 4 μg/L [30]. The level of fusidic acid resistance in the isolate 4 with two fusidic acid resistance determinants couldn't be accounted for by their genotypes when compared with other clinical isolates with one of the determinants. A previous study showed a similar result that a laboratory strain containing both *fusA *resistance mutation and *fusB *failed to increase the level of fusidic acid resistance [17]. The chromosomal gene *fusC *confer resistance to fusidic acid on *S. aureus *or *S. intermedius *is identified with 45% amino acid similarity to FusB, protect EF-G from the antibiotic [18]. Genes for FusB-type resistance (*fusB *and *fusC*) are thought to act by the same mechanism of protection the drug target [18]. It remains unclear whether these resistance mechanisms of a strain do act in combination or not. The precise action mode of FusB-type resistance awaits further investigation. The level of fusidic acid resistance in isolate 32 did not decrease after curing the pUB101 plasmid. The result may indicate that the resistance mechanisms do not act synergistically or additively.

In this study, all MRSA isolates met the criteria of being health-care associated. PFGE patterns revealed that there was greater than 80% similarity among the isolates. MLST and SCC*mec *typing showed that all isolates belonged to ST239 and carried SCC*mec *III elements, which is the most prevalent health care-associated strain of MRSA in Taiwan [31]. A previous study conducted in 2002-2007 in northern Taiwan also revealed that most of fusidic acid-resistant MRSA isolates carried SCC*mec *type III [27]. The two studies results suggest that a clonal strain had disseminated in Taiwan during the period of the study. In contrast to our findings, a previous European study finding indicated that the majority of fusidic acid-resistant MRSA isolates belonged to CC80-MRSA-IV clone carrying *fusB *and CC5 clone harbouring *fusC *[30].

## Conclusion

In conclusion, we hypothesize that the prevalence of fusidic acid-resistance in *S. aureus *was commonly associated with the *fus*C determinant in our isolates. It is interesting to note that some studied isolates possessed more than one fusidic acid-resistance mechanism in our collection. The *fusC *and acquired FusB-family determinants in a single isolate were first detected and one isolate with *fusC *also carried a *fus*A mutation in H_457_Y. Phylogenetic analysis clearly demonstrated the spread of a major clonal strain of fusidic acid-resistant MRSA in our institution. Due to the concern of clonal spread and growing expansion of fusidic acid-resistant determinants, particularly FusC in MRSA, large-scale, prospective surveillance monitoring for fusidic acid-resistance in *S. aureus *and MRSA is now ongoing in Taiwan.

## Abbreviations

BSAC: British Society of Antimicrobial Chemotherapy; CLSI: Clinical and Laboratory Standards Institute; EUCAST: European Committee for Antimicrobial Susceptibility Testing; FA: fusidic acid; IRB: Institutional Review Board; LB: Luria-Bertani; MICs: minimum inhibitory concentrations; MLST: multilocus sequence typing; MRSA: methicillin-resistant *Staphylococcus aureus*; MSSA: methicillin-susceptible *S. aureus*; PCR: polymerase chain reaction; PFGE: pulsed field gel electrophoresis; SCC: staphylococcal chromosome cassette; SCVs: small-colony variants; TTMHH: Tungs' Taichung MetroHarbor Hospital.

## Competing interests

The authors declare that they have no competing interests.

## Authors' contributions

CMC planned the idea and prepared the manuscript. MH participated in the study design and provided resources of experimental work. HFC conducted the experimental work. SCK and CRL provided technical help with PFGE and MLST. JHW supervised study design. LTW conceived this study, participated in its design, and the coordination and writing of the manuscript. All authors read and approved the final manuscript.
